# Photobiomodulation ameliorates inflammatory parameters in fibroblast-like synoviocytes and experimental animal models of rheumatoid arthritis

**DOI:** 10.3389/fimmu.2023.1122581

**Published:** 2023-03-29

**Authors:** Ji Hyeon Ryu, Jisu Park, Bo-Young Kim, Yeonye Kim, Nam Gyun Kim, Yong-Il Shin

**Affiliations:** ^1^ Research Institute for Convergence of Biomedical Science and Technology, Pusan National University Yangsan Hospital, Yangsan, Republic of Korea; ^2^ Medical Research Center of Color Seven, Seoul, Republic of Korea; ^3^ Department of Rehabilitation Medicine, School of Medicine, Pusan National University, Yangsan, Republic of Korea

**Keywords:** LED therapy, photobiomodulation, collagen-induced arthritis, rheumatoid arthritis, NOD-like receptor family pyrin domain containing 3, NF-kappa B

## Abstract

**Introduction:**

Rheumatoid arthritis (RA) is a chronic destructive inflammatory disease that afflicts over one percent of the world’s population. Current pharmacological treatments remain relatively ineffective. In this context, photobiomodulation (PBM) is a potential resource for the treatment of RA. This study investigates investigate the anti-arthritic effects and related mechanisms of PBM on fibroblast-like synoviocytes (FLSs) from RA patients and a mouse model of collagen-induced arthritis (CIA).

**Methods:**

The RA-FLSs were irradiated with a light emitting diode (LED) at a wavelength of 610 nm for 20 min, and the corresponding power intensities were 5 and 10 mW/cm^2^. After the LED irradiation, cell viability, proliferation, migration, and invasion assays were performed. Male DBA/1J mice were used to establish an animal model of CIA. Light stimulation with 10 mW/cm^2^ was applied to the ankle joints *via* direct contact with the skin for 40 min, daily for 2 weeks.

**Results and Discussion:**

PBM significantly reduced tumor necrosis factor (TNF)-α-induced increase in proliferation, migration, and invasion in RA-FLSs, and downregulated the activation of nuclear factor-κappa B (NF-κB) and NLRP3 inflammasome by TNF-α. Moreover, PBM greatly inhibited the induction and development of CIA, resulting in the inhibition of synovial inflammation and cartilage degradation. PBM therapy decreased the serum levels of pro-inflammatory cytokines, while increasing the anti-inflammatory cytokines. PBM suppressed the translocation of NF-κB and activation of NLRP3 inflammasome in the ankle joint. Furthermore, PBM showed a more pronounced anti-arthritic effect when combined with methotrexate (MTX), a disease-modifying anti-rheumatic drug (DMARD). The results showed that the effectiveness of MTX + PBM in CIA is superior to that of either MTX or PBM and that both work synergistically. Therefore, PBM with LED may be a potential therapeutic intervention for against RA.

## 1 Introduction

Rheumatoid arthritis (RA) is a systemic autoimmune disease characterized by dysregulated inflammation of the synovium and destruction of bone and cartilage in multiple peripheral joints. Although the precise pathogenesis of RA remains unclear, systemic inflammatory responses appear to be closely associated with the development and progression of RA. The synovium is the primary site of the inflammatory process, and fibroblast-like synoviocytes (FLSs) are key effector cells in RA. During the progression of RA, constant inflammatory responses occur in the synovial membrane; RA-FLSs show apoptosis resistance, excessive proliferation, migration, invasion and release of pro-inflammatory mediators, resulting in the destruction of bone and cartilage (1).

RA affects approximately 0.5–1% of the population worldwide and causes high disability and mortality rates (2). The major clinical signs and symptoms of RA include warm and swollen joints, joint stiffness that is usually worse in the mornings and after inactivity, fatigue, fever, loss of appetite, and limitations in daily life activities (3, 4). These features may reduce the quality of life of RA patients. Currently, RA treatment employs several medications, either in combination or alone; glucocorticoids, nonsteroidal anti-inflammatory drugs, disease-modifying anti-rheumatic drugs (DMARDs), and biologics are the main drugs commonly prescribed to treat RA patients (5). In particular, methotrexate (MTX), one of the DMARDs, is the most commonly administered drug for moderate and severe RA, although long-term treatment with high-doses of MTX has been reported to cause systemic adverse effects (6). Furthermore, a significant proportion of RA patients still report poor response rates, infection, and high therapeutic costs, often restricting the prescription of this drug (7, 8). Therefore, developing new, safer, less toxic, and more effective therapeutic strategies for RA is extremely urgent.

Laser therapy has the potential to enhance wound healing and alleviate pain, inflammation, and swelling. More recently, the field, sometimes known as photobiomodulation (PBM), has been broadened to include light emitting diodes (LED) and other light sources, and the range of wavelengths now used includes many in the red and near infrared spectral region. PBM is a therapeutic intervention widely used by physiotherapists, dentists, and physicians to treat many ailments, primarily due to its antioxidant, anti-inflammatory, and analgesic properties (9–11). It is also used to maximize the effectiveness of medications. It elicits a non-thermal response wherein, light interacts with chromophores leading to photophysical and photochemical reactions in different tissues, thereby promoting the modulation of cell metabolism (12). Though LED was introduced as an electronic component in 1962, its modern versions are used for therapeutic purposes and are available at different wavelengths, such as blue, near-infrared, red, orange, and green (13). PBM treatment with LED is a non-invasive, non-painful, inexpensive, easy-to-use, and safe therapeutic intervention, without side effects even after prolonged use. PBM has been proposed as a therapeutic modality for arthritis owing to its antioxidant (14), anti-inflammatory (15, 16), and analgesic effects (10). The other beneficial reasons are relief from edema (16), restoration of cartilage integrity (17), and inhibition of abnormal proliferation of human synoviocytes (18) and chondrosarcoma cells (19). Although the beneficial effects are known, the underlying mechanisms through which they occur are not fully understood.

This study investigates the anti-arthritic effects and related mechanisms of PBM using a 610 nm LED, and the synergistic effect of the simultaneous administration of MTX and PBM, to degermine their therapeutic potential for treating RA.

## 2 Materials and methods

### 2.1 Cell culture and light emitting diode-based photobiomodulation (PBM) irradiation

An LED-based device (width × length × height: 4.9 × 4.9 × 1.3 cm, Color Seven Co., Seoul, Korea) was used for PBM with the following parameters: wavelength, 610 nm [full width at half maximum, 24 nm (orange color)]; power intensity, 5 and 10 mW/cm^2^; energy density, 12 J/cm^2^; surface area of electrodes, 1.6 cm; spot size of electrodes, 4 mm diameter.

Fibroblast-like synoviocytes from RA patients (RA-FLSs) were purchased from Cell Applications (San Diego, CA, USA) and cultured in synoviocyte Growth Medium (Cell Applications) at 37°C with 5% CO_2_. The medium was replaced every two days. For PBM treatment, RA-FLSs were seeded in triplicate, at a density of 5 × 10^4^ cells/well in 24-well plates (SPL Life Sciences Co. Ltd., Pocheon, Korea). After 18 h, the culture medium was changed to serum-free medium, and the cells were exposed to 10 ng/mL of tumor necrosis factor-α (TNF-α, Quimigen, Madrid, Spain) and/or PBM at 610 nm wavelength for 20 min in the dark; the corresponding power intensity were 5 and 10 mW/cm^2^, respectively. Control cells were treated in the same manner, except for the light exposure. Subsequently, the cells were collected and tested for viability, proliferation, migration, invasion, and Western blot analysis.

### 2.2 Cell viability

Cell viability of RA-FLSs was determined colorimetrically using the 3-(4,5-dimethylthiazole-2-yl)-2,5-diphenyl tetrazolium bromide (MTT) reagent (Duchefa, Haarlem, Netherlands). The MTT assay procedure was performed as previously described (20). Briefly, RA-FLSs were seeded in 24‐well plates (5 × 10^4^ cells/well) and cultured at 37°C with 5% CO_2_ for 24 h before LED irradiation. On the following day, the culture medium was replaced with a serum-free medium, and cells were irradiated with LED light for 20 min. After 24 h, MTT solution (50 μL of 5 mg/mL) was added to each well and incubated for an additional 2 h at 37°C in the dark. The supernatants were removed, and the formazan crystals in each well were dissolved in 500 μL of dimethyl sulfoxide for 30 min at room temperature while shaking. The absorbance was measured at 570 nm using a microplate reader (Tecan, Infinite M200, Austria). Data are expressed as the percentage of viable cells in comparison with the control.

### 2.3 Cell proliferation

Cell proliferation was determined using 5-bromo-2′-deoxyuridine (BrdU; Sigma-Aldrich, St. Louis, MO, USA) incorporation as described previously (21). Briefly, RA-FLSs were grown on a round cover glass coated with poly-L-lysine (Sigma-Aldrich) for 24 h at density of 1 × 10^5^ cells/mL in 24-well plates. After 18 h, the culture medium was replaced with serum-free medium, and cells were exposed to LED at 610 nm for 20 min. On the following day, RA-FLSs were incubated at 37°C for 3 h with 10 μM of BrdU. The cells were fixed with 4% paraformaldehyde for 30 min, washed three times with PBS, and permeabilized *via* incubation in 0.2% Triton X-100 in PBS for 30 min. To measure BrdU incorporation, cells were incubated in 2 M hydrochloric acid for 30 min at 37°C to denature the DNA, then incubated for an additional 5 min in 0.1 M sodium borate (pH 8.5) to neutralize the residual acid and washed three times with PBS. Thereafter, the cells were incubated in a blocking solution containing 1% normal goat serum for 1 h at room temperature and with monoclonal anti-BrdU (1:100 dilution, Thermo Fisher Scientific, Waltham, MA, USA) in a blocking solution at 4°C overnight. After washing with PBS thrice, the cells were incubated with Alexa Fluor 488 goat anti-mouse IgG (1:500 dilution, Thermo Fisher Scientific, Rockford, IL, USA) in the dark for 1 h. BrdU-positive cells were counted in five distinct fields per slide and expressed as a percentage of the total cells counted. Total DNA was determined and quantified using propidium iodide (PI).

### 2.4 Cell migration

Migration assays were performed as described previously (22). Briefly, a culture-insert (ibidi GmbH, Martinsried, Germany) was attached to 24-well plates. Cells (7.5 × 10^5^ cells/mL; 70 μL) were seeded into each well and incubated for 24 h in the culture medium with 25 μg/mL mitomycin C (Sigma) for 30 min to inhibit cell division and proliferation. The culture-insert was taken out, cells were irradiated, and migration was analyzed after 0, 24, and 48 h. The migration of cells into the cell-free gap created by the removal of the culture-insert was monitored at the indicated time points and photographed using a microscope (Nikon, Tokyo, Japan). The velocity of migration was determined through a quantitative assessment. All data obtained are from at least three independent experiments performed in triplicate. The Transwell migration assay was performed using the CytoSelectTM Cell Migration Assay Kit containing polycarbonate membrane inserts (8 μm pore membrane; Cell Biolabs, San Diego, CA, USA) according to the manufacturer’s instructions and as previously described (22). Briefly, RA-FLSs (2 × 10^4^ cells) were resuspended in serum-free medium, placed in the upper chamber, and allowed to migrate for 12 h. The lower chamber, containing medium with 10% FBS, allowed cell migration towards the lower face of the trans-well culture inserts. Cells were irradiated with LED for 20 min and incubated for 12 h at 37°C with 5% CO_2_. Non-migrating cells on the inner side of the trans-well culture inserts were gently removed using a cotton-tipped swab. The migrated cells on the bottom surface were stained with 0.1% crystal violet solution (Sigma) for 15 min. Photomicrographs of three individual fields per insert were obtained using a microscope (Nikon) and analyzed using ImageJ software (version 1.49, National Institutes of Health, Bethesda, MD, USA) to calculate the average number of migrated cells.

### 2.5 Cell invasion

The cell invasion assay was performed using a Transwell system (Corning Incorporated, Corning, NY, USA) as previously described (22). The 8 µm pore size polycarbonate membrane was coated with Matrigel (BD Biosciences, San Jose, CA, USA). The cells (2 × 10^4^) were suspended in 300 µL serum-free medium and added to the upper chamber, while 500 µL medium containing 10% FBS was added to the lower well of each chamber. After 24 h of incubation at 37°C, the invading cells in the lower chambers were stained with 0.1% crystal violet solution for 15 min, and counted using a microscope (Nikon).

### 2.6 Induction of collagen-induced arthritis (CIA) and PBM irradiation

Male DBA/1J mice, aged 7–8 weeks, were obtained from Central Lab. Animal Inc. (Seoul, Korea). The mice were housed in a pathogen-free facility and provided food and water *ad libitum*. All experiments were approved by the Institutional Animal Care and Use Committee of Pusan National University, in accordance with the National Institutes of Health Guidelines (PNU-2020-2757).

CIA was induced as previously described (23). Mice models were immunized intradermally on day 0 at the base of the tail with 100 μg bovine type II collagen (CII; Chondrex Inc., Redmond, WA, USA), emulsified with an equal volume of complete Freund’s adjuvant (CFA; Sigma, St. Louis, Mo. USA). Immunization was boosted with an equal volume of emulsion of CII emulsion and incomplete Freund’s adjuvant (IFA; Chondrex) on day 21. At the time of CII injection, normal mice were immunized and boosted with PBS. Methotrexate (MTX; Sigma), a medicine used for RA, was administered intraperitoneally once every 3 days, from day 22 to day 35. After boosting, the mice were divided into five groups (*n* = 7–8) as follows: normal, CIA, CIA + MTX (1 mg/kg every 3 days), CIA + PBM, and CIA + MTX + PBM. Light stimulation at 10 mW/cm^2^ was applied to the ankle joints *via* direct contact with the skin for 40 min daily from day 21 to day 35 under isoflurane (Hana Pharm Co., Ltd., Hwaseong, Korea; 2% induction and 1.5% maintenance in 80% N_2_O and 20% O_2_) anesthesia. The normal, CIA, and CIA+MTX groups were anesthetized for 40 min without PBM.

### 2.7 Clinical assessment of arthritis

Mice were closely monitored and scored 3 times a week after the primary collagen injection and were scored independently by three investigators for each limb on a 4-point scale according to the following criteria (23): 0 = normal paw; 1 = erythema and mild swelling confined to the tarsal or ankle joint; 2 = erythema and mild swelling extending from the ankle to the tarsal; 3 = erythema and moderate swelling extending from the ankle to the metatarsal joints; 4 = erythema and severe swelling encompassing the ankle, foot, and digits, or ankylosis of the limb. The scores of all four paws were summed up to obtain the arthritis score.

### 2.8 Histopathologic examination

The mice were sacrificed on day 36, and the ankle joints were collected and fixed in 10% phosphate- buffered formaldehyde solution for 24 h. The joints were decalcified in RDO-Gold decalcifier (Electron Microscopy Sciences, Hatfield, PA, USA) for 3 days and dehydrated and embedded in paraffin. Tissue sections (thickness, 3 μm) were prepared and stained with hematoxylin and eosin (H&E; Sigma-Aldrich) and safranin-O (Sigma-Aldrich). Synovial inflammation and hyperplasia were determined using H&E staining according to the following criteria: 0, no signs of inflammation; 1, slight thickening of the lining layer or some infiltrating cells in the underlying layer; 2, slight thickening of the lining layer plus some infiltrating cells in the underlying layer; 3, thickening of the lining layer, an influx of cells in the underlying layer, and the presence of cells in the synovial space; and 4, synovium highly infiltrated with many inflammatory cells. Cartilage damage was determined using safranin-O staining according to the following criteria: 0, no destruction; 1, minimal erosion limited to single spots; 2, slight-to-moderate erosion in a limited area; 3, more extensive erosion; 4, general destruction. Stained specimens were visualized using a virtual microscope (Axio Scan.Z1; Carl Zeiss, Heidenheim, Germany). Histological assessment was performed by three blinded, independent observers, and the results were indicated as the average of the observer’s scores.

### 2.9 Determination of superoxide anion

Histological determination of superoxide anions in the ankle joint was performed with dihydroethidium (DHE; Thermo Scientific) staining. Briefly, deparaffinized tissue sections were incubated with 10 μM DHE in PBS for 30 min at 37°C in a humidified chamber protected from light. The sections were washed twice with PBS, visualized using a confocal microscope (LSM 900, Carl Zeiss, Germany).

### 2.10 Determination of total free radical activity

Total free radical activity was measured using the OxiselectTM *In Vitro* ROS/RNS assay kit (Cell Biolabs, San Diego, CA, USA) as previously described (24).

### 2.11 Micro-computed tomography (CT) analysis

Computed tomography images of the hind paws of the mice in all five groups (*n* = 4) were acquired on day 36 using a micro-CT (Quantum FX, Perkin Elmer, MA, USA). The ankle joints of the experimental groups were scanned a tube voltage of 90 kV, tube current 160 uA, 20 um resolution and 2 min scan time.

### 2.12 Enzyme-Linked Immunosorbent Assay (ELISA)

Mouse anti-CII antibodies (IgG, IgG1, and IgG2a; Chondrex Inc., Redmond, WA, USA), mouse TNF-α, interleukin (IL)-6, IL-17, IL-10, transforming growth factor (TGF)-β, IL-1β/12 (R&D Systems, Minneapolis, MN, USA), IL-18 (Abcam, Cambridge, UK), and TRACP 5b (CUSABIO Biotech., Wuhan, Hubei, China) were measured using ELISA kits according to the manufacturer’s instructions. The absorbance was read at 450 nm using a microplate reader (Tecan; Infinite™ M200 PRO; Männedorf, Switzerland).

### 2.13 RNA extraction and quantitative real-time polymerase chain reaction (PCR)

Total RNA was isolated from the joint tissue using the TRIzol reagent (Invitrogen, Carlsbad, CA, USA), and cDNA was synthesized using the amfiRivert Platinum cDNA synthesis master mix (GenDEPOT, Barker, TX, USA) according to the manufacturer’s instructions. Real-time PCR was performed using a Rotor-Gene Q real-time PCR system (Qiagen, Hilden, Germany) with a FastStart Essential DNA Green Master (Roche Diagnostics, Mannheim, Germany). Expression of target genes was calculated using the 2−ΔΔCt comparative method for relative quantification after normalization against glyceraldehyde 3-phosphate dehydrogenase (GAPDH). The primer sequences (forward and reverse) were as follows: *Trap*, 5′-CCAATGCCAAAGAGATCGCC-3′ and 5′-TCTGTGCAGAGACGTTGCCAAG-3′; *Ctsk*, 5′-GACGCAGCGATGCTAACTAA-3′ and 5′- CCAGCACAGAGTCCACAACT-3′; *Ctr*, 5′-TCAGGAACCACGGAATCCTC-3′ and 5′-ACATTCAAGCGGATGCGTCT-3′; *Mmp-9*, 5′-CTGGACAGCCAGACACTAAAG-3′ and 5′-CTCGCGGCAAGTCTTCAGAG-3′.

### 2.14 Tissue fraction and western blot analysis

Western blotting was performed as described previously (25). The cytosolic and nuclear fraction preparation was performed using ExKine™ Nuclear and Cytoplasmic Protein Extraction Kit (Abbkine, China) according to the manufacturer’s instructions. The primary antibodies used were as follows: anti-NLRP3 (1:1,000; Cell Signaling, Danvers, MA, USA), anti-XIAP (1:1,000; Enzo Life Sciences, Farmingdale, NY, USA), anti-ASC (1:1,000; Abbkine), anti-caspase-1 (1:1,000; Novus Biologicals), anti-TLR4 (1:1,000; Bioworld Technology), anti-myeloid differentiation factor88 (MyD88, 1:1,000; Cell Signaling), anti-NF-κB (1:1,000; Cell Signaling), anti-Lamin B1 (1:1,000; Cell Signaling), anti-β-tubulin (1:1,000; Abbkine), and anti-β-actin (1:5,000; Enzo Life Sciences). The membranes were then washed thrice with Tris-buffered saline with Tween-20 (TBST) and incubated with secondary antibodies conjugated with horseradish peroxidase. Immunopositive bands were enhanced with chemiluminescence (Amersham Pharmacia, Piscataway, NJ, USA) and visualized using the Fusion Solo X (Vilber Lourmat, Collegien, France).

### 2.15 Statistics

Data are expressed as the mean ± standard error (SE). The one-way ANOVA/Bonferroni test or the Kruskal–Wallis/Mann–Whitney test was selected after the normality test to analyze differences among groups (OriginPro2020, OriginLab Corp., Northampton, MA, USA). A *p* < 0.05 was considered significantly different.

## 3 Results

### 3.1 PBM alleviated TNF-α-induced increase in proliferation, migration, and invasion in RA-FLSs

**Figure 1:**
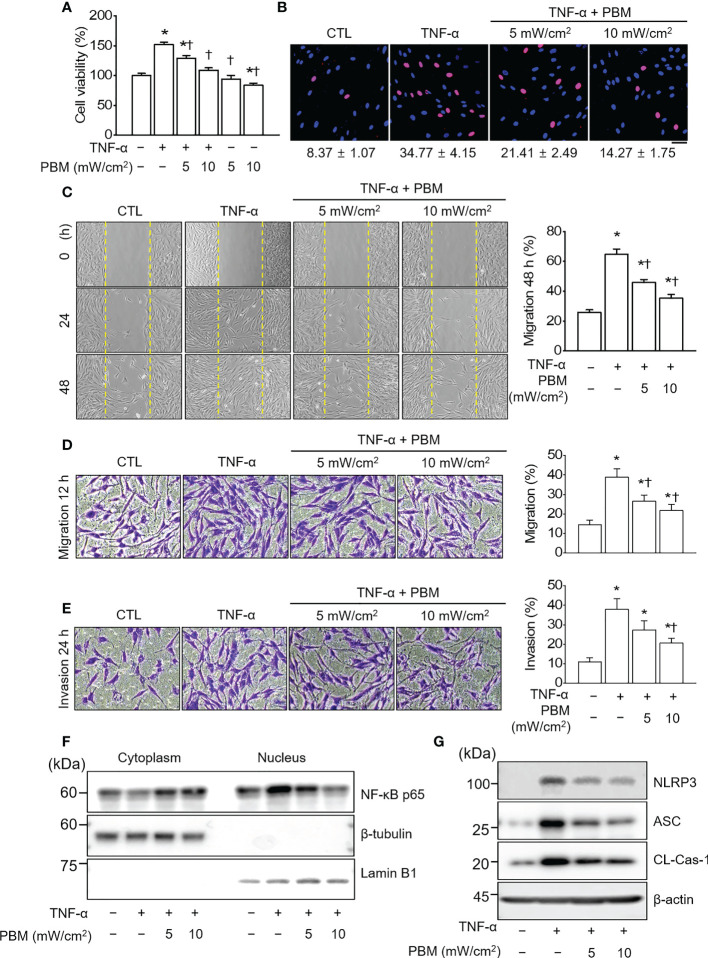
PBM suppresses TNF-α-induced proliferation, migration, and invasion in RA-FLSs. **(A)** Effect of PBM on RA-FLSs cell viability (*n* = 4). Cells were exposed to 10 ng/mL of tumor necrosis factor (TNF)-α and/or PBM at 610 nm wavelength for 20 min with power intensities of 5 and 10 mW/cm^2^. After 24 h, cell viability was measured by 3-(4,5-dimethylthiazole-2-yl)-2,5-diphenyl tetrazolium bromide (MTT) assay as described in the Materials and Methods 2.2. **(B)** RA-FLSs were treated with 10 ng/mL of TNF-α and/or PBM at 610 nm wavelength for 20 min with power intensities of 5 and 10 mW/cm^2^ (*n* = 3). After 24 h, 5‐Bromo‐2′‐deoxyuridine (BrdU) immunofluorescence staining was performed as described in the Materials and Methods 2.3. Immunocytochemistry showed localization of BrdU-labeled nuclear (red) and nuclear staining (blue). Bar graphs represent the quantification of BrdU-positive cells. Scale bar, 30 μm. **(C)** Representative phase-contrast images at 0, 24, and 48 h showing the migration of RA-FLSs into the cell-free gap left by the removal of the Culture-Insert (*n* = 3). Bar graphs represent the percentage of migrated cells 48 h after PBM treatment. **(D)** Representative microscopic images of migrating cells by transwell migration assays (*n* = 3). **(E)** Representative microscopic images of invasive cells by transwell system (*n* = 3). **(F)** Detection of NF-κB p65 in cytoplasmic and nuclear fractions. Cell lysates were fractionated into nucleus and cytoplasm components (*n* = 2). Nucleus fraction was confirmed by expression of lamin B1, a nuclear structural protein, and the absence of expression of β-tubulin. **(G)** Detection of NLRP3 inflammasome (*n* = 3). Data are shown as the mean ± SE. ^*^ *p* < 0.05, compared to CTL. ^†^ *p* < 0.05, compared to TNF-α.

To generate an *in vitro* inflammatory system for investigating the protective effects of PBM, RA-FLSs were stimulated with TNF-α (10 ng/mL) in the presence or absence of 610 nm LED irradiation at intensities of 5 and 10 mW/cm^2^ for 20 min. TNF-α significantly increased the cell viability in RA-FLSs as compared with that observed in the control (*p* < 0.05; **Figure 1A**); the increase in cell viability was reduced substantially by PBM. The TNF-α induced increase in cell viability was reduced more by LED irradiation with 10 mW/cm^2^ than with 5 mW/cm^2^ (*p* < 0.05). Moreover, LED irradiation of cells treated with 10 mW/cm^2^ alone significantly reduced the cell viability as compared with that of the control (*p* < 0.05). To assess the proliferation of RA-FLSs after LED irradiation, the cells were labeled with BrdU. The proliferation in the TNF-α-induced RA-FLSs was significantly enhanced as compared with that in the control (*p* < 0.05; **Figure 1B**). However, the enhanced proliferation caused by TNF-α was suppressed by PBM in an intensity-dependent manner (*p* < 0.05). To determine whether PBM regulated RA-FLSs migration, a migration assay using a silicon-based culture insert and transwell chamber was performed. The TNF-α treatment significantly increased the cell migration by more than 2-fold compared with that in the control group at 48 h after treatment (*p* < 0.05; **Figure 1C**). However, pretreatment with 5 mW/cm^2^ and 10 mW/cm^2^ significantly reduced the TNF-α-induced cell migration by 29.19 ± 5.39% and 38.85 ± 7.43%, respectively (*p* < 0.05). To further confirm these results, we then carried out experiments using transwell chambers and obtained similar results (**Figure 1D**). TNF-α significantly increased cell migration in RA-FLSs at 24 h after treatment, which was markedly attenuated by PBM therapy (*p* < 0.05). To determine the effect of PBM on RA-FLSs cell invasion, a transwell invasion assay was performed. A significantly increased number of TNF-α-treated RA-FLSs were observed on the membranes of the transwell chamber, compared with the control (*p* < 0.05; **Figure 1E**); The cell invasion was significantly decreased in the LED irradiation with 10 mW/cm^2^ group. To determine the molecular mechanism underlying the inhibitory effects of PBM on cell proliferation, migration, and invasion in RA-FLSs, we investigated the effect of PBM on the activation of NF-κB and NLRP3 inflammasome pathways in RA-FLSs. Cells were irradiated with LED light for 20 min before stimulation with TNF-α (10 ng/mL) for 30 min. The nuclear translocation of NF-κB and NLRP3 inflammasome activation were observed in RA-FLSs stimulated by TNF-α and were suppressed by PBM (**Figures 1F, G**). These results suggested that PBM inhibited TNF-α-stimulated proliferation, migration, and invasion of RA-FLSs through regulation of NF-κB and NLRP3 inflammasome activation.

### 3.2 PBM improved clinical arthritic condition in CIA mice

**Figure 2:**
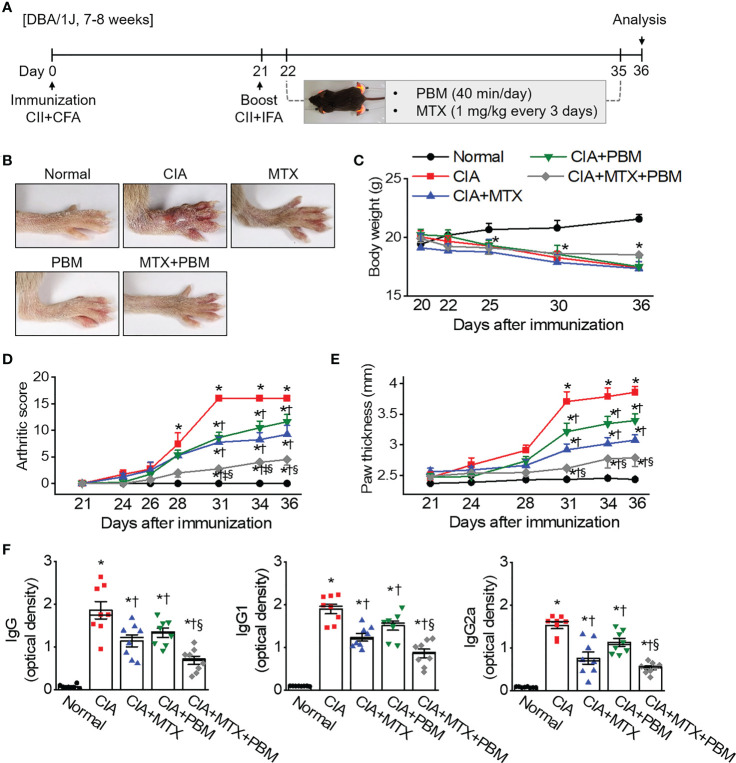
PBM alleviates the clinical symptoms of collagen-induced arthritis (CIA) in DBA/1J mice. **(A)** Production of CIA mouse model. Schematic representation of experimental procedure. Mice were immunized on Day 0 with intradermal injection of 100 μg of bovine type II collagen (CII) emulsified with an equal volume of complete Freund’s adjuvant (CFA). Immunization was boosted by an equal volume of emulsion of CII and incomplete Freund’s adjuvant (IFA) on Day 21. At the time of CII injection, normal mice were immunized and boosted with PBS. Methotrexate (MTX) as a positive control was administered intraperitoneally once every 3 days from day 22 to day 35. Light stimulation with 10 mW/cm^2^ was applied to the ankle joints *via* direct contact with the skin for 40 min daily from day 21 to day 35. Animal experiments were performed in triplicate. **(B)** Gross observation of the hind paw; photographs are representative of each group on Day 36. **(C)** Body weight was measured on the indicated days after the primary immunization. **(D)** Mean arthritic score for each group. The severity was evaluated on a scale from 0 to 4. The scores of all four paws were summed to obtain the arthritis score. **(E)** Paw thickness was measured using an electric caliper by three researchers independently. **(F)** Collagen-specific antibodies in the mouse sera were measured using the enzyme-linked immunosorbent (ELISA). Data are shown as mean ± SE (*n* = 7−8). ^*^ *p* < 0.05, compared to Normal. ^†^ *p* < 0.05, compared to CIA. ^‡^ *p* < 0.05, compared to CIA+MTX. ^§^ *p* < 0.05, compared to CIA+PBM.

To investigate the *in vivo* role of PBM on RA, we used a well-established CIA mouse model of human RA. DBA/1J mice were employed and the results of PBM were compared with those of methotrexate (MTX), a medication typically used in the treatment of RA. PBM treatment was initiated on day 22 after the first immunization. The severity of arthritis was assessed by observation, every 3 days after the booster injection (**Figure 2A**). The CIA mice treated with the vehicle displayed severe inflammatory responses, including redness and swelling in the paw, as compared with the normal mice (**Figure 2B**). Conversely, the inflammatory responses in the paw were significantly reduced in the CIA mice treated with PBM as compared with those treated with the vehicle control. The body weight of the mice in the normal group showed a steady increase (**Figure 2C**). The CIA mice showed severe body weight loss as compared with the normal mice, but no significant differences were evident among the four collagen-sensitized groups. Compared with the CIA group, the CIA + MTX or CIA + PBM groups showed a significantly lower arthritis score and paw thickness during the entire experimental period (*p* < 0.05; **Figures 2D, E**). Moreover, the combination of MTX and PBM exhibited a greater therapeutic effect than MTX or PBM alone (*p* < 0.05). The total CII-specific IgG, IgG1, and IgG2a serum levels were determined in the experimental groups. CIA mice showed a significant increase in the CII-specific IgG, IgG1, and IgG2a serum levels as compared with the normal group; the increase was attenuated by PBM (*p* < 0.05; **Figure 2F**). The MTX and PBM combination therapy showed the maximum decrease in the production of the total CII-specific IgG, IgG1, and IgG2a serum levels. Collectively, these data suggested that PBM exerted therapeutic effects in CIA mouse model *in vivo*.

### 3.3 PBM attenuates synovial inflammation and cartilage destruction

**Figure 3:**
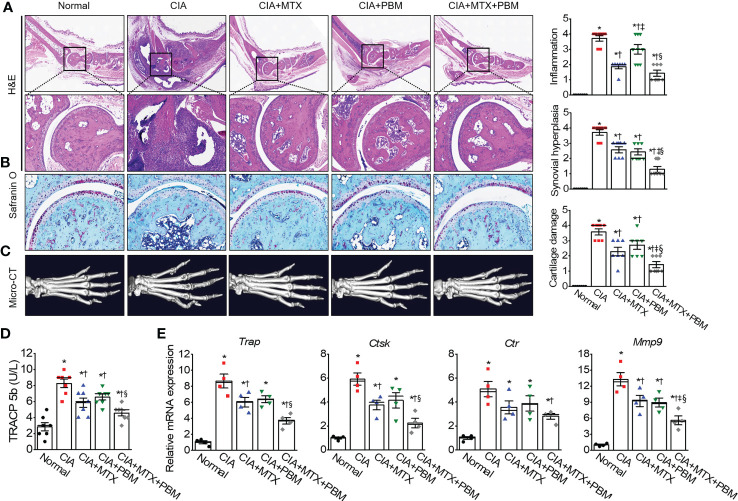
PBM mitigates histopathological abnormalities in CIA mice. **(A)** Histological analysis of 3 μm sections of paraffin-embedded ankle joints tissue stained with hematoxylin and eosin (H&E). Photographs are of representatives from each experimental group. Degree of synovial inflammation and hyperplasia was evaluated on a scale from 0 to 4. Representative images of ankle joints tissue stained with **(B)** Safranin-O in each experimental group. Degree of cartilage damage was evaluated on a scale from 0 to 4 (*n* = 7). **(C)** Representative photographs of 3D reconstruction of micro-computed tomography of the hind paws of mice with CIA. **(D)** The serum level of bone marker TRACP 5b in each group (*n* = 7). **(E)** The relative mRNA of osteoclastogenesis-related markers in paws were detected by real-time PCR (*n* = 4). Data are shown as mean ± SE. ^*^ *p* < 0.05, compared to Normal. ^†^ *p* < 0.05, compared to CIA. ^‡^ *p* < 0.05, compared to CIA+MTX. ^§^ *p* < 0.05, compared to CIA+PBM.

To examine the therapeutic effects of PBM on arthritic inflammation and articular joint destruction in CIA mice, sections of the ankle joints and paws were prepared from the five experimental groups. The CIA mice revealed histopathological changes, including marked infiltration of inflammatory cells into synovial tissues, synovial hyperplasia, and loss of articular cartilage and bone (**Figures 3A, B**). Compared with the CIA mice, both MTX and PBM treatments significantly attenuated the pathological symptoms of CIA in the ankle joint. Furthermore, the combination of MTX and PBM exhibited a more pronounced therapeutic effect than that exerted by MTX or PBM alone in treated mice. A micro-CT scan of the hind paws showed articular destruction in the CIA mice; these were observed alleviated to a significant extent in the both MTX and PBM groups. Moreover, the combination of MTX and PBM blocked the bone destruction of the hind paws more effectively as compared to that observed in the MTX or PBM group (**Figure 3C**).

The serum levels of TRAPC 5b, related to bone erosion, were measured in the experimental groups (**Figure 3D**). CIA mice showed an increase in the TRAPC 5b serum levels as compared with the normal mice; this increase was suppressed by PBM (*p* < 0.05) and the suppression was greater when PBM was combined with MTX (*p* < 0.05). The CIA mice showed an increase in the expression of all the four osteoclast-specific genes, such as *Trap*, *Ctst*, *Ctr*, and *mmp9* mRNA, as compared with those in the normal mice (*p* < 0.05, **Figure 3E**); this increase was significantly reversed by the combined treatment with MTX + PBM (*p* < 0.05). These results suggested that PBM maintained normal bone structure in mice. Thus, PBM treatment reduced the inflammation and prevented damage to the bone and cartilage tissues in the ankle joints in CIA mice.

### 3.4 PBM attenuated NF-κB-mediated inflammatory response in CIA mice

**Figure 4:**
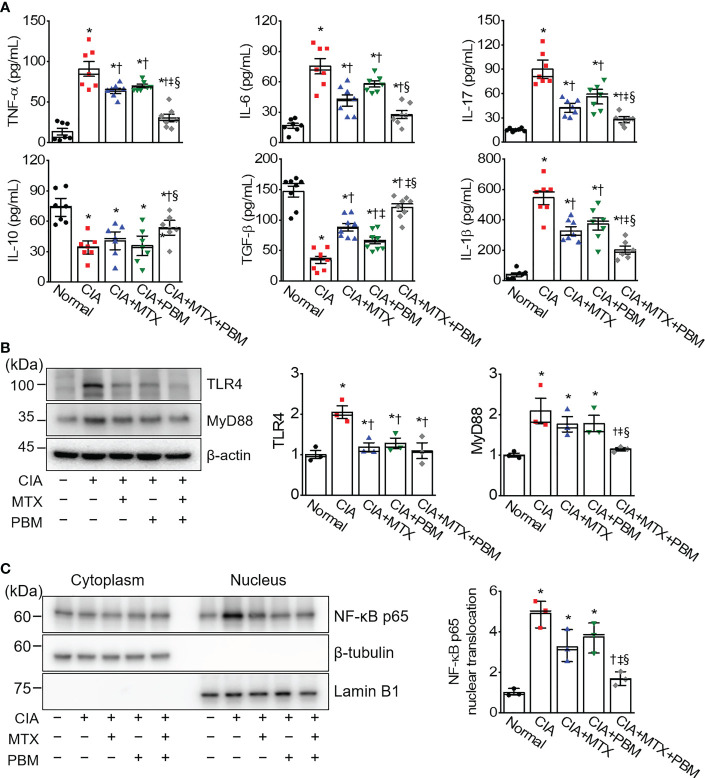
PBM reduces TLR4/NF-κB-mediated inflammation in CIA mice. **(A)** Measurement of TNF-α, IL-1β, IL-6, IL-17, IL-10, and TGF-β in serum (*n* = 7). **(B)** Expression of TLR4 and MyD88 in the ankle joints tissue lysates (*n* = 3). **(C)** Detection of NF-κB p65 in cytoplasmic and nuclear fractions. Ankle joints tissue lysates were fractionated into nucleus and cytoplasm components (*n* = 3). Nucleus fraction was confirmed by expression of lamin B1, a nuclear structural protein, and the absence of expression of β-tubulin. An aliquot of cell lysate was analyzed by immunoblotting. Data are shown as mean ± SE. ^*^ *p* < 0.05, compared to Normal. ^†^ *p* < 0.05, compared to CIA. ^‡^ *p* < 0.05, compared to CIA+MTX. ^§^ *p* < 0.05, compared to CIA+PBM.

To verify the effect of PBM on the inflammatory responses in CIA mice, the serum levels of the various cytokines collected from the five experimental groups were measured using ELISA (**Figure 4A**). In CIA mice, there was a significant increase in the level of TNF-α, IL-1β, IL-6, and IL-17, and decrease in the secretion of IL-10 and TGF-β (*p* < 0.05). However, a concomitant decrease in the level of pro-inflammatory cytokines and an increase in anti-inflammatory cytokines was observed in the serum of PBM-treated mice as compared with the CIA mice (*p* < 0.05). Moreover, the levels of pro-inflammatory cytokines were observed to be the lowest and those of anti-inflammatory cytokines were the highest in the CIA + MTX + PBM treatment group. These results suggested that PBM has the potential to suppress the production of pro-inflammatory cytokines and induce anti-inflammatory cytokines.

To determine whether PBM protected against inflammation in CIA mice through the TLR4/MyD88/NF-κB pathway, we investigated the expression levels of TLR4, MyD88, and NF-κB in the ankle joint tissue of CIA mice using immunoblotting. The immunoblotting results showed that TLR4 and MyD88 protein expression in the ankle joints in the CIA group was more pronounced as compared with the normal group. In comparison with the CIA group, the protein expression levels of TLR4 and MyD88 were markedly decreased in the CIA + MTX + PBM treatment group (**Figure 4B**). The activation of NF-κB was evaluated in CIA-induced ankle joints tissue. Nuclear NF-κB translocation was observed to be greater in the CIA group as compared with that in the normal mice (**Figure 4C**), while the translocation was inhibited in both CIA + MTX and CIA + PBM groups, but the difference was not significant. However, the combination of MTX and PBM inhibited the NF-κB translocation significantly (*p* < 0.05).

### 3.5 PBM inhibits the activation of ROS-mediated NLRP3 inflammasome in CIA mice

**Figure 5:**
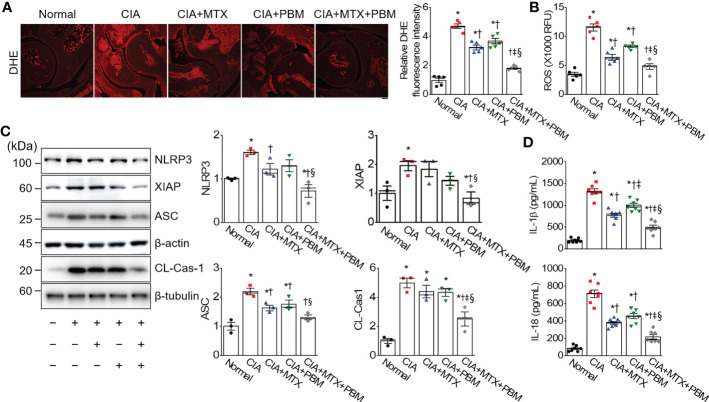
PBM inhibites ROS-mediated inflammasome activation in CIA mice. **(A)** Superoxide [dihydroethidium (DHE)] staining in ankle joints tissue. Scale bar, 100 μm. Bar graphs represent the quantification of DHE fluorescence intensity. **(B)** ROS level. RFU represents relative fluorescence units (*n* = 5). **(C)** Detection of pyroptosis signals (*n* = 3). **(D)** Measurement of IL-1β and IL-18 in ankle joints tissue lysates (*n* = 7). Data are shown as mean ± SE. ^*^ *p* < 0.05, compared to Normal. ^†^ *p* < 0.05, compared to CIA. ^‡^ *p* < 0.05, compared to CIA+MTX. ^§^ *p* < 0.05, compared to CIA+PBM.

ROS are potential signals for NLRP3 inflammasome formation and activation (26). To investigate the regulation of ROS production in CIA mice by PBM, the levels of superoxide anion were detected by using DHE staining in the ankle joint tissue section. DHE fluorescence intensity in ankle joint tissue was increased in the CIA group as compared with the normal group, but showed a significant decrease in the CIA + PBM group (*p* < 0.05; **Figure 5A**). To further confirm the observation, the ROS production was examined by measuring the mean fluorescence intensity of DCF. In the CIA group, the levels of ROS in the ankle joint tissue lysates was high in comparison with the normal group (**Figure 5B**). The ROS levels showed a significant decrease of 45% and 28% in the CIA + MTX and CIA + PBM groups, respectively (*p* < 0.05). In addition, combined therapy with MTX and PBM showed the maximum decrease in the production of ROS (58% of the CIA group, *p* < 0.05).

The expression levels of NLRP3 inflammasome-related proteins, including NLRP3, X-linked inhibitor of apoptosis (XIAP), apoptosis-associated speck-like protein containing a caspase recruitment domain (ASC), and cleaved caspase-1 were investigated in the ankle joint tissue of CIA mice using immunoblotting. The expressions of NLRP3, XIAP, and cleaved caspase-1 were observed to be higher in the ankle joint of CIA mice than those of the normal group; however, these changes were markedly reversed by treatment with CIA + MTX + PBM (*p* < 0.05; **Figure 5C**). To confirm the inhibition of NLRP3 inflammasome activation, the levels of pro-inflammatory cytokines produced by the NLRP inflammasome, IL-1β and IL-18, were measured in the ankle joint lysate. PBM significantly reduced the levels of mature IL-1β and IL-18 in the ankle joint tissue compared with those in the CIA group (*p* < 0.05; **Figure 5D**). The above findings suggested that the TLR4/MyD88/NF-κB signaling participated in the inflammatory response of CIA mice and promoted NLRP3 inflammasome activation.

## 4 Discussion

The present study demonstrated that PBM, with LED at a wavelength of 610 nm, effectively attenuated the TNF-α induced acceleration in cell proliferation, migration, and invasion in RA-FLSs in a power intensity-dependent manner. PBM also suppressed the nuclear translocation of NF-κB and activation of the NLRP3 inflammasome by TNF. PBM treatment, every day for 14 days, was observed to remarkably alleviate the arthritis symptoms in CIA mice by reducing the synovial inflammation and cartilage destruction, along with a decrease in the production of autoantibodies and pro-inflammatory cytokines in the serum. Moreover, administration of a combination of MTX and PBM to the CIA mice resulted in a pronounced synergistic effect as compared with that seen with MTX or PBM alone. PBM inhibited the activation of the nucleotide-binding domain (NOD)-like receptor 3 (NLRP3) inflammasome, a result consistent with the suppression of pro-inflammatory cytokines in the ankle joint through the inhibition of TLR4-mediated NF-κB signaling.

MTX has been used since the 1950s to treat RA and is currently the most widely used DMARD. MTX reduces the rate of joint destruction by participating in a series of immune responses initiated by the inflammatory cytokines and joint-destroying enzymes (27). Despite these effects, the exact mechanism, other than the treatment mechanism of RA through the inhibition of cell proliferation by MTX, has not been identified, and there are concerns about the systemic side effects of long-term administration. From this point of view, non-pharmacological interventions, such as PBM, cannot completely replace MTX, but by using PBM together with MTX, the dose of the drug can be reduced and the symptomatic relief can be maximized.

Recently, the use of PBM therapy with lasers or LEDs has received attention as a treatment intervention for RA in contrast to the prolonged administration of nonsteroidal anti-inflammatory drugs and analgesics, which only control the symptoms and have serious adverse effects. The beneficial effects of PBM on arthritis have been previously illustratede by Martins et al., wherein PBM with 630 nm LED showed positive effects in recovering from oxidative stress and in preserving the articular cartilage aspects in a knee osteoarthritis (OA) rat model (14). In addition, PBM with 850 nm LED was reported to suppress degenerative processes and stimulate the immune reactivity of TGF-β and collagen type 2 in knees OA rats (28). PBM with 940 nm LED was effective in inhibiting the clinical symptoms and inflammatory reactions in CIA mice (16). PBM using laser effectively preserved the glycosaminoglycan content (29), reduced the inflammatory processes in human chondrocytes (29, 30), and promoted cartilage recovery in monoiodoacetate (MIA)-induced OA (17). PBM with laser at 680 and 830 nm significantly inhibited the expression of the inflammatory genes, edema, vascular permeability, and hyperalgesia; however, treatment with 628 nm LED, with the same fluence as the laser, showed no effect on zymosan-induced arthritis (15). Our results showed that PBM treatment effectively attenuated the aggravation of arthritis manifestation and suppressed the anti-CII antibody production in CIA mice. Histological examination and micro-CT scan of the hind paws further confirmed that synovial hyperplasia and cartilage damage were ameliorated by the PBM therapy. Moreover, the combined use of MTX and PBM improved synovial inflammation and bone loss when compared with the use of MTX or PBM alone. Our results on the beneficial effects of PBM on arthritis are consistent with the previous studies mentioned above; however, the light source used for PBM, the wavelengths, power intensity, exposure duration, number and frequency of treatment sessions, and application techniques differ.

The FLSs are one of the major cell types located in the inner layer of the synovial membrane, are a major source of the synovial fluid, and are critical for maintaining homeostasis of the internal joints. Activated RA-FLSs have been found to share features with cancer cells, such as excessive proliferation, and aggressive cell behavior, including enhanced secretion of inflammatory and chemotactic cytokines, and increased migratory and invasive abilities; as a result, activation of RA‐FLSs during inflammation contributes to the formation of rheumatoid pannus, which promotes the initiation and development of RA (1). TNF-α, a pivotal pro-inflammatory cytokine associated with RA, can stimulate the proliferation of FLSs and increase the production of inflammatory mediators (31). In this study, TNF-α was used to stimulate human RA‐FLSs to generate an *in vitro* inflammatory environment. Our results showed that PBM with 610 nm LED significantly suppressed the TNF-α-stimulated cell proliferation, migration, and invasion of RA‐FLSs. Consistent with our results, PBM with red LED reportedly suppressed pro-inflammatory cytokine-induced cell proliferation in human synoviocyte MH7A cells (18) and in human chondrosarcoma cell SW1353 (19). In another study on the fibroblasts of the anterior cruciate ligament of New Zealand white rabbits, blue LED (460 nm) showed cytotoxicity; while red (630 nm) and green LED (530 nm) promoted cell proliferation and migration, and increased expression of integrin, growth factors, and extracellular matrix without cellular toxicity (32). These findings imply that the protective effects of PBM on RA may be through suppression of the abnormal proliferation, migration, and invasion of RA-FLSs.

Pyroptosis, a form of programmed cell necrosis, is emerging as a general innate immune signal and is accompanied by the activation of the inflammasome and the maturation of pro-inflammatory cytokines (33). The NLRP3 inflammasome consists of NLRP3, apoptosis-associated speck-like protein containing a caspase recruitment domain (ASC), precursor caspase-1, and precursor caspase-11 or X-linked inhibitor of apoptosis (XIAP), or both (34). It can be activated by numerous physically and chemically diverse stimuli, including microbes, K^+^ efflux or Ca^2+^ signaling, ATP, endosomal rupture, and ROS generation (35). IL-1β and IL-18 persist in damage to endothelial cells, which exacerbates synovial inflammation and cardiovascular disease in RA patient (34). IL-18 is involved in the inflammation by inducing leukocyte extravasation *via* an increase in endothelial cell adhesion molecules, induction of chemokine release from RA-FLSs, and by acting as a monocyte, lymphocyte, and neutrophil chemoattractant that thereafter induces bony erosion (36). The NLRP3 inflammasome is primarily involved in the pathogenesis and development of RA, and the polymorphisms of NLRP3 gene have been associated with RA susceptibility and pathological severity (37). The levels of NLRP3, caspase-1, and IL-1β were reportedly higher in the peripheral blood cells of RA patients (38) and CIA mice (39). Suppression of NLRP3 with a highly selective NLRP3 inhibitor, MCC950, was effective in ameliorating arthritis symptoms and cartilage erosion in CIA mice (39). Excessive activation of NLRP3 inflammasome drives the pathogenesis of arthritis in spontaneous arthritis animal models (A20 ^myel-KO^ mice) (40).

Evidence suggests that NLRP3 is activated by TLR4 signaling, resulting in the formation of intracellular inflammasome complexes with the adaptor protein ASC, and caspase-1-dependent cleavage and release of IL-1β (38). Synovial TLR4 was observed to be higher in RA patients and found to positively correlate with synovitis (41). In CIA and K/BxN serum transfer model, TLR4 deficient mice were found protected from joint destruction and showed reduced inflammatory cell infiltration (42, 43). NF-κB signaling was a necessary prerequisite for the proper activation of the NLRP3 inflammasome. NF-κB regulates the expression of the NLRP3 gene and plays a critical role in priming the NLRP3 inflammasome (44). IL-1β leads to the activation of the NF-κB signaling cascade to induce cytokine production, which results in the transcriptional activation of the genes encoding chemokines and various pro-inflammatory mediators (45). Disruption of the NF-κB/NLRP3 connection alleviates RA injury in CIA mice (46) and cartilage degradation in an osteoarthritic rat model (47). In this study, the NLRP3/TLR4/NF-κB signaling pathways were activated in our model; these were reversed by PBM treatment. This shows that the inhibitory effect of PBM on the inflammatory response is multifaceted.

Mitochondrial cytochrome c oxidase (Cco) is considered a major photoreceptor of photosignals and catalyzes nitric oxide (NO) synthesis (48). The Cco is a primary source of cellular NO *via* catalyzation nitrite (NO_2_^–^)-dependent NO synthesis; and this NO_2_^–^ reductase activity of Cco is referred to as Cco/NO (49). NO regulates several humoral and cellular responses in inflammation, having both anti-inflammatory and pro-inflammatory properties depending on the type and phase of the inflammatory reaction (50). The activity of Cco/NO peaks at the wavelength range of the orange color (590–630 nm) (48). Therefore, we hypothesized that a wavelength of 610 nm, the center value of the orange color wavelength range, would induce anti-inflammatory effects in RA. The LED parameters used in this study reduced the characteristic manifestations of RA while acting positively on the efficacy of MTX, but this may not necessarily translate into direct use for RA patients.

The selection of sub-optimal parameters may result in reduced effectiveness or even cause negative treatment results (51). To establish accurate treatment mechanisms for specific ailments, quantification and standardization of light parameters, such as wavelength, irradiation time, power density, pulse structure, and the timing of the applied light should be possible, and the effectiveness of scientific clinical trials must be verified. Based on our findings, we suggest that PBM may be a suitable nonpharmacological intervention for RA that may reduce RA symptoms and provide potential treatment, even though we still need more detailed studies.

In conclusion, PBM with 610 nm LED showed anti-rheumatic effects and affected inhibition in cell proliferation, migration, and invasion in RA-FLSs, and in paw thickness, autoantibodies, synovial inflammation, hyperplasia, cartilage damage, pro-inflammatory cytokines, ROS, pyroptosis signals, and TLR4-mediated NF-κB activation in mouse models of CIA. Moreover, PBM showed a more effective anti-arthritic effect when combined with MTX. These results suggest that PBM is a potential non-pharmacological interventional therapeutic strategy for the treatment of RA.

## Data availability statement

The original contributions presented in the study are included in the article/supplementary material. Further inquiries can be directed to the corresponding author.

## Ethics statement

The study was conducted in accordance with the guidelines of the Declaration of Helsinki and approved by the Animal Care and Use Committee of Pusan National University.

## Author contributions

JR and YK established the animal models and collected the samples. JR, YK, JP, and B-YK analyzed and interpreted the data. JR wrote the manuscript. NK participated in the research design and interpretation of the data. Y-IS supervised the project. All authors contributed to the article and approved the submitted version.
